# Lineage-Specific Expansion of the Chalcone Synthase Gene Family in Rosids

**DOI:** 10.1371/journal.pone.0133400

**Published:** 2015-07-16

**Authors:** Kattina Zavala, Juan C. Opazo

**Affiliations:** Instituto de Ciencias Ambientales y Evolutivas, Facultad de Ciencias, Universidad Austral de Chile, Valdivia, Chile; Fordham University, UNITED STATES

## Abstract

Rosids are a monophyletic group that includes approximately 70,000 species in 140 families, and they are found in a variety of habitats and life forms. Many important crops such as fruit trees and legumes are rosids. The evolutionary success of this group may have been influenced by their ability to produce flavonoids, secondary metabolites that are synthetized through a branch of the phenylpropanoid pathway where chalcone synthase is a key enzyme. In this work, we studied the evolution of the chalcone synthase gene family in 12 species belonging to the rosid clade. Our results show that the last common ancestor of the rosid clade possessed six chalcone synthase gene lineages that were differentially retained during the evolutionary history of the group. In fact, of the six gene lineages that were present in the last common ancestor, 7 species retained 2 of them, whereas the other 5 only retained one gene lineage. We also show that one of the gene lineages was disproportionately expanded in species that belonged to the order Fabales (soybean, barrel medic and *Lotus japonicas*). Based on the available literature, we suggest that this gene lineage possesses stress-related biological functions (e.g., response to UV light, pathogen defense). We propose that the observed expansion of this clade was a result of a selective pressure to increase the amount of enzymes involved in the production of phenylpropanoid pathway-derived secondary metabolites, which is consistent with the hypothesis that suggested that lineage-specific expansions fuel plant adaptation.

## Introduction

Gene duplication has been considered a fundamental process in providing raw genetic material in evolution [1, 2]. Extra gene copies can be originated in several ways, from small-scale events via unequal crossing over or retrotransposition to large-scale events, including segmental or whole genome duplications. Episodes of whole genome duplications have characterized the evolutionary history of plants, and most of them occurred around the Cretaceous-Paleogene boundary [3]. They have played an important role during the evolution of plants because they can even be responsible for the generation of new species [4].

Angiosperms are a major plant group and are likely the most diverse group with approximately 260,000 classified living species in approximately 453 families [5, 6]. Among them, rosids are a monophyletic group comprising nearly 70,000 species in 140 families [6–8]. Many important crops, such as fruit trees and legumes, are rosids. They are characterized by unique biochemical properties, such as symbiotic machinery with nitrogen-fixing bacteria [6]. The evolutionary success of some rosid families may be partially due to their flavonoid production. Flavonoids are a key group of plant secondary metabolites that are synthetized through a branch of the phenylpropanoid pathway. Different flavonoid classes have been associated with fundamental plant functions, such as pathogen defense, which is particularly important in legumes (isoflavonoids), UV light protection (flavonols), and plant pigmentation (anthocyanins) [9, 10]. Each of these processes is important for the evolutionary success of plants, and therefore natural selection likely plays a significant role in shaping the evolutionary history of enzymes involved in this biochemical pathway.

**Fig 1:**
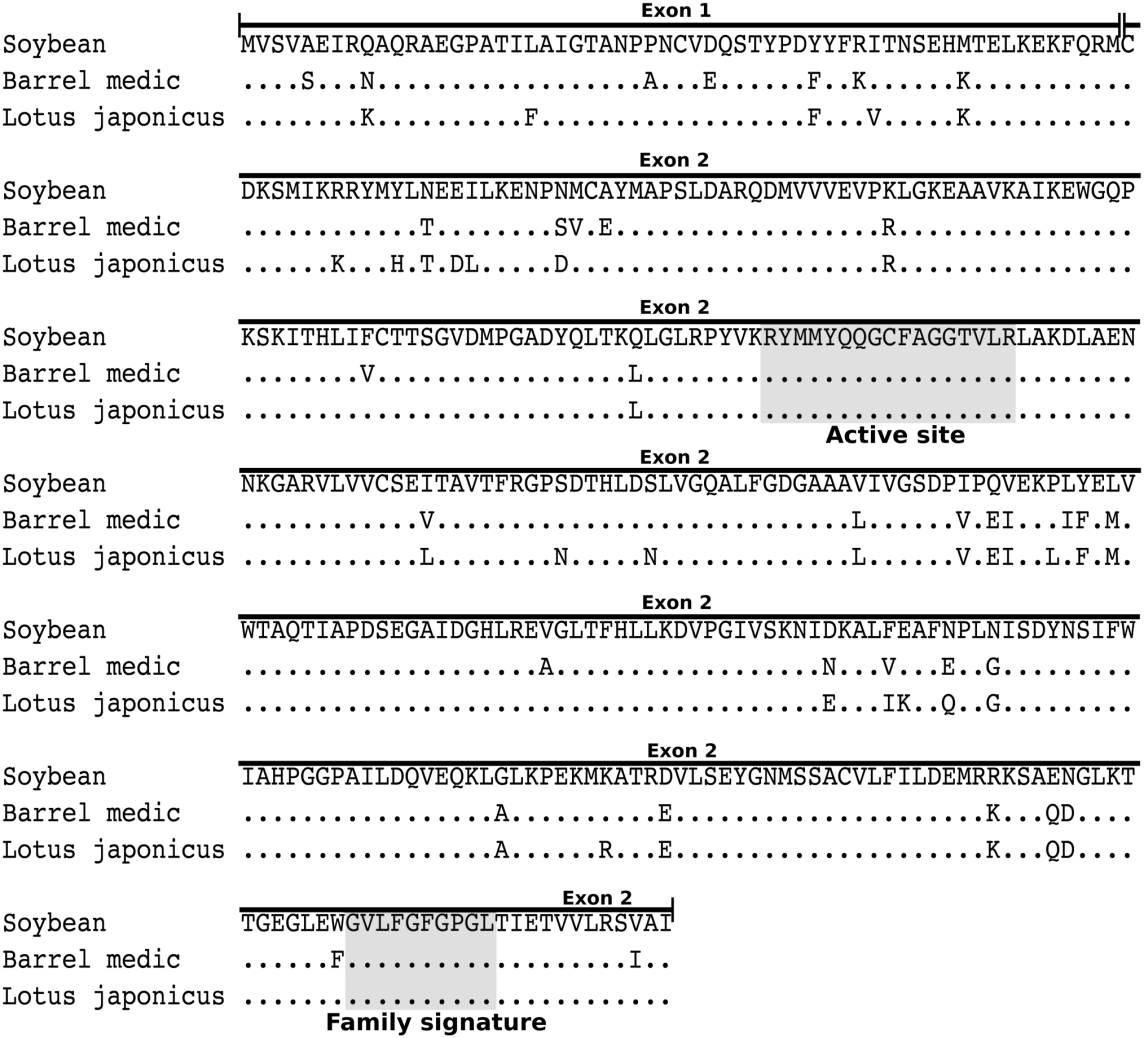
Alignment of chalcone synthase amino acid sequences from soybean, barrel medic and *Lotus japonicus*. Sequences correspond to the gene found in chromosome 11, 1 T1 and 2 T5, respectively. Shaded areas represent the amino acid sequence that defines the molecule’s active site and the defining amino acid sequence of the gene family.

Chalcone synthase (CHS; EC 2.3.1.74) is a key enzyme in flavonoid biosynthesis and is encoded by the chalcone synthase gene family, which is a member of a superfamily that also includes stilbene synthase (STS), acridone synthase (ACS), 2-pyrone synthase (2-PS), bibenzyl synthase (BBS), and coumaroyl triacetic acid synthase (CTAS) [11]. CHS catalyzes the condensation of three acetate residues from malonyl-CoA with *p*-coumaroyl-CoA to form naringenin chalcone [12]. This is the initial step of the phenylpropanoid pathway that leads to flavonoid production. Comparison of CHS gene sequences from different species revealed that the CHS gene is structurally conserved and contains two exons separated by one intron. The first exon is less conserved, and it varies in length from 37 to 64 amino acids. The second exon is more conserved and encodes 340 residues. The amino acid sequence that defines the molecule’s active site and the amino acid sequence that defines the signature of the gene family reside in the second exon (Fig 1) [13–15]. The number of CHS genes varies among species; for example, thale cress (*Arabidopsis thaliana*) possesses one CHS gene [16, 17], whereas soybean has 7 CHS genes.

The aim of this work was to study the evolution of the chalcone synthase gene family in representative species of the rosid clade. We performed phylogenetic analyses to characterize the duplicative history of chalcone synthase genes in 12 species of the rosid group. The results of our evolutionary analyses revealed that the last common ancestor of the rosid clade possessed six CHS gene lineages that were differentially retained during its evolutionary history. We also found that one gene lineage disproportionately expanded in species belonging to the order Fabales. Based on the available literature, we suggest that this gene lineage possesses stress-related biological functions (e.g., UV light response, pathogen defense).

## Materials and Methods

### DNA data

The chalcone synthase (CHS) genes from 12 species of flowering plants belonging to the rosid clade were manually annotated. DNA sequences from structural genes were obtained from the Ensembl Plant database (release 21). CHS genes were identified by comparing known exon sequences with genomic fragments using the blast2seq program, version 2.2 [18] available from NCBI (http://www.ncbi.nlm.nih.gov/blast/bl2seq). PLAZA 2.5-derived sequences (http://bioinformatics.psb.ugent.be/plaza/) were also included to attain broad and balanced taxonomic coverage (S1 Table). The species included in this study comprised three Brassicales (thale cress, lyrate rockcress and field mustard), one Malvaceae (cacao), three Malphigiales (cassava, castor bean and poplar), two Rosales (apple and wild strawberry) and three Fabales (barrel medic, *Lotus japonicus* and soybean). Putatively functional genes were characterized by an intact open reading frame with the canonical CHS gene structure of two exons and one intron. Because CHS genes have undergone multiple rounds of duplication resulting in the presence of sets of paralogous gene copy tandem repeats, we indexed each duplicated gene with the symbol T followed by a number that corresponds to the linkage order in the 5’ to 3’ orientation. Pseudogenes were indexed with the ps suffix.

### Phylogenetic inference

Phylogenetic relationships among CHS genes were estimated using a maximum likelihood approach, as implemented in the Treefinder version March 2011 [19] and CodonPhyML programs [20]. The latter approach uses a more realistic description of the evolutionary process at the protein-coding sequence level by incorporating the genetic code structure into the model. Nucleotide translated sequences were aligned using the L-INS-i strategy from MAFFT v.6 [21]. Nucleotide sequences were aligned using the amino acid alignment as a template in the TranslatorX software (http://translatorx.co.uk/; [22]) (S1 File). Best fitting models for each codon position were separately estimated using the propose model routine from the Treefinder program, version March 2011 [19] (S2 File). In the case of maximum likelihood using Treefinder, we estimated the best tree under the selected models (S3 File) and assessed support for the nodes with 1,000 bootstrap pseudoreplicates. In the maximum likelihood approach implemented in the CodonPhyML program [20], the model described by Goldman & Yang using a subtree pruning and regrafting (SPR) heuristic search with 5 random starting trees, was used to reconstruct phylogenetic relationships [23]. Support for the nodes was assessed following the aBayes method [24]. The choice of an adequate outgroup is important to have a reliable phylogenetic tree which will allow us to recognize gene lineages and to define pathways of evolution within the group of interest. In this study we included LAP5 (Less Adhesive Pollen 5) and STS (stilbene synthase) gene sequences as outgroups, given that they are members of the same superfamily [11].

## Results and Discussion

We studied the evolution of the chalcone synthase gene family in a sample of flowering plants including representative species of the orders Brassicales, Malvaceae, Malpighiales, Rosales and Fabales. We manually annotated CHS genes in the representative species’ genomes for which genomic information was available. We reconstructed pathways of gene family evolution using a phylogenetic approach, from which we can conclude that diversity observed in extant species is the result of a combination of different evolutionary processes where gene sorting played an important role.

**Fig 2:**
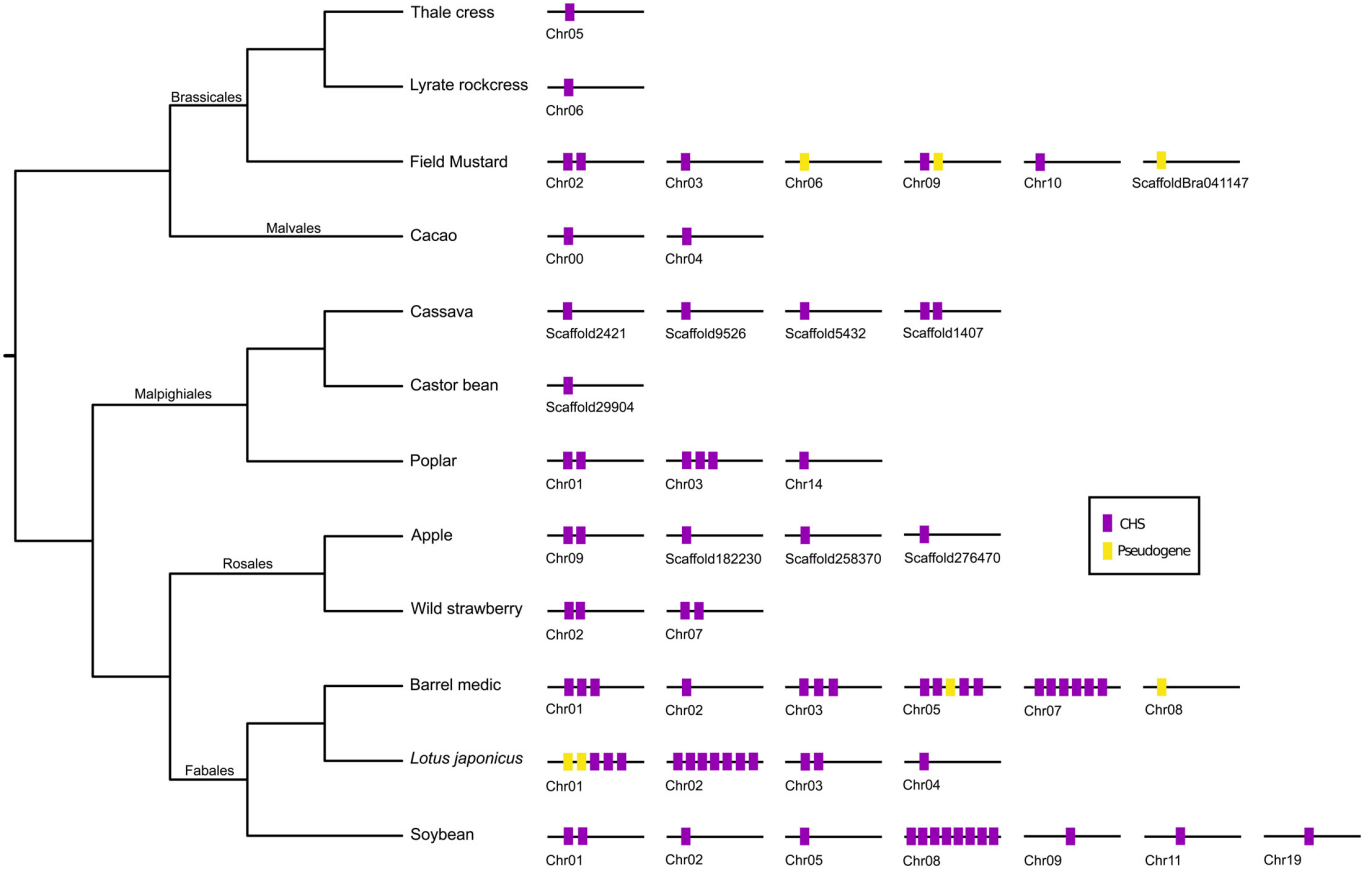
Genomic structure of the chalcone synthase gene family in rosids. The orientation of the genomic pieces (chromosomes/scaffolds) is from 5′ (on the left) to 3′ (on the right).

Our results show that the number of putatively functional CHS genes varies among species, ranging from one in thale cress, lyrate rockcress and castor bean to 17 in the barrel medic (Fig 2). Our results were consistent with previous reports of thale cress, in which one CHS gene was identified [16], but are inconsistent with reports for other species [25]. A total of 8 to 12 gene copies have been previously reported for the barrel medic [26], whereas 7 CHS genes were identified in soybean [27]. In contrast, we identified 17 and 15 CHS genes, respectively (Fig 2). Aside from putatively functional genes, we also identified pseudogenes in three species (Fig 2). We identified two pseudogenes in the barrel medic and *Lotus japonicus* and three in field mustard (Fig 2). Pseudogenes were recognizable because the portion of the second exon containing the gene family signature and the active site was present (Fig 1). We also found that the number of chromosomes in which CHS genes were present also varied (Fig 2). According to our results, the number of chromosomes containing CHS genes ranged from one in thale cress, lyrate rockcress and castor bean, to seven in soybean (Fig 2). The distribution of paralogs on different chromosomes and the number of genes on each chromosome can be interpreted as a balance between multiple rounds of whole genome duplication during the group’s evolutionary history [3], the gene turnover dynamic after whole genome duplications [28], and the rate of gene movement among chromosomes. Our results show that the interaction of these factors resulted in a variable pattern (Fig 2). For example, although the clade containing two *Arabidopsis* species has experienced two tetraploidy events during the rosid clade evolution, they only possess one putatively functional CHS gene. On the other hand the clade containing the barrel medic and *Lotus japonicus* species underwent one tetrapolidy event, during the rosid clade’s evolutionary history and possesses a more diverse gene repertoire (Fig 2).

Gene phylogenies allow us to define gene lineages and to infer the repertoire of genes in the last common ancestor of our group of interest. Gene lineages can be defined as sets of genes that can trace their evolutionary origin to a common ancestral gene, and the reconstruction of the gene complement of the ancestor of interest relies in interpreting gene phylogenies in the context of organismal phylogenies. In principle, for a given set of genes from a group of organisms with a known phylogeny, gene phylogenies would allow us to identify gene lineages and to infer how many of them can be traced back to the last common ancestor of our group of interest. In the simplest case, in the absence of gene losses and duplications, a gene lineage is defined as a clade that contains gene copies from all species included in the study and the gene phylogeny ideally matches the organismal phylogeny. However, this is rarely the situation, as genomes gain and lose genes over time. In most cases, not all species retain representative genes from all lineages. Here, a simple situation could be given when the clade does not contain all surveyed species, however, the phylogenetic representation of them includes all main groups of the organismal phylogeny. In this case it is safe to say that this gene lineage was present in the last common ancestor of the group (e.g. green, brown and red clades). Alternatively, when the species repertoire of the clade does not represent all main groups of the organismal phylogeny, it would imply that the gene was present in the last common ancestor of the group and lost in all other species (e.g. pink clade). An extreme case is when a gene is retained by a single species (e.g. orange and blue clades), which might also imply that the gene was present in the ancestor and lost in all other species. Gene gains and losses are pervasive and can occur multiple times along a given gene phylogeny, making this a challenging problem.

**Fig 3:**
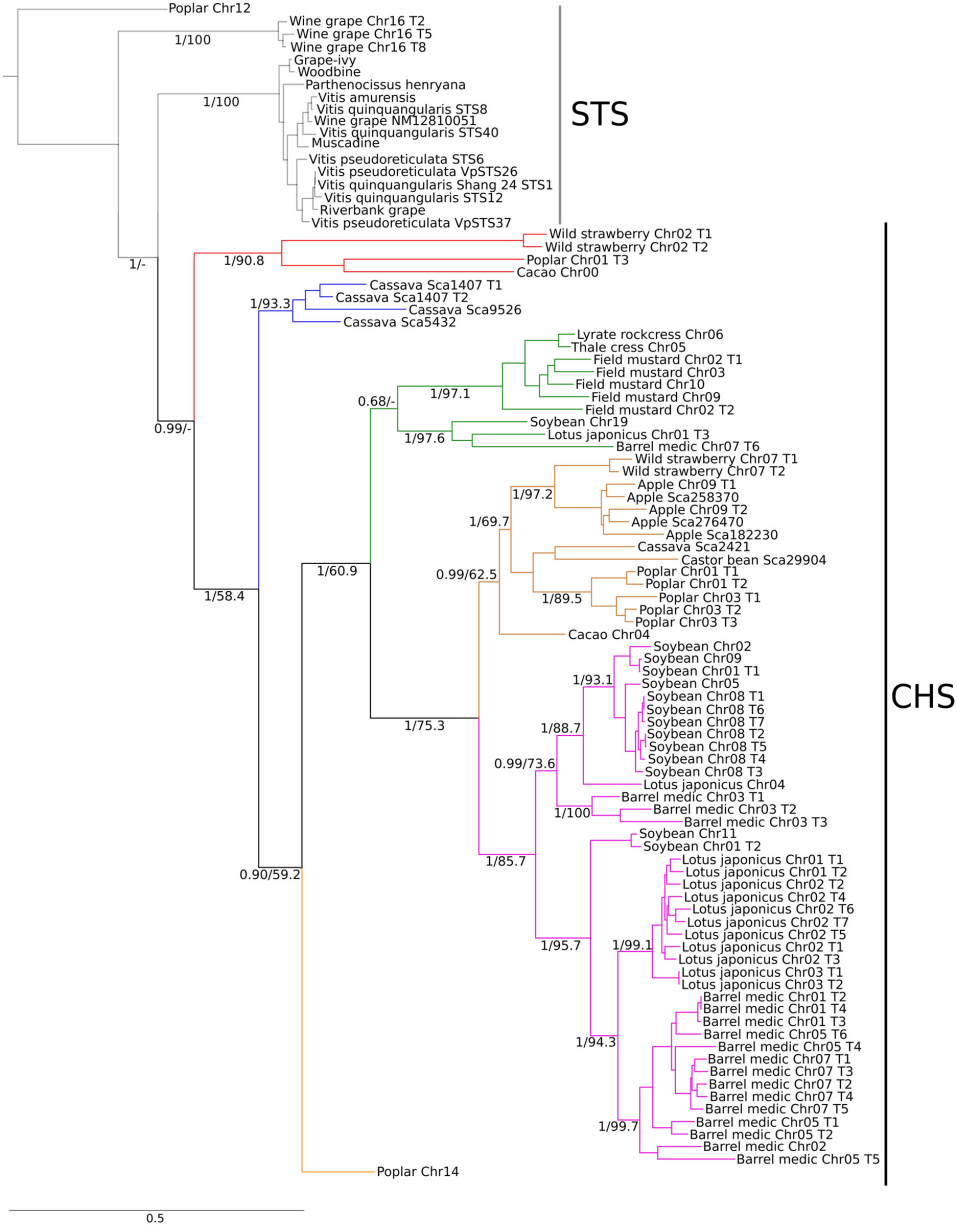
Maximum likelihood phylogram depicting relationships among chalcone synthase genes. Sequences of LAP5 (Less Adhesive Pollen 5) (not shown) and STS (stilbene synthase) were included as outgroups. The numbers on the nodes correspond to the posterior probability using the aBayes approach (left) and the maximum likelihood bootstrap support (right).

According to our phylogenetic analyses, the CHS gene diversity is derived from an ancestral repertoire of six CHS gene lineages present in the last common ancestor of the rosid clade, approximately 107 mya (Fig 3). This repertoire was inherited and differentially retained by different species in the group. Our results show that 7 species retained 2 of the six gene lineages from the last common ancestor of the rosid clade, whereas the other 5 species only possess one gene lineage (Fig 3). After this process, lineage-specific dynamics gave rise to the observed gene repertoire in each species (Fig 2). According to our analyses, different gene lineages possess different numbers of gene copies, ranging from one (yellow lineage) to 42 gene copies (pink lineage) (Fig 3). Additionally, genes were retained by a variable number of species (Fig 3), ranging from one (blue and yellow lineages) to six species (brown lineage) (Fig 3).

The most restricted gene lineages are the blue and yellow lineages, which were retained by one species each, the cassava and poplar, respectively (Fig 3). In the blue lineage, several rounds of gene duplication gave rise to the four-gene repertoire in the cassava (Fig 3). The red clade was retained by three rosid species representative of the orders Malvales (cacao), Malpighiales (poplar) and Rosales (wild strawberry). A gene duplication event gave rise to a second copy of the gene in the wild strawberry (Fig 3). The green clade was retained by six species, three of the order Brassicales (thale cress, lyrate rockcress and field mustard) and three of the order Fabales (barrel medic, *Lotus japonicus* and soybean) (Fig 3). In this clade, our phylogenetic analyses suggest that a duplication event gave rise to an extra gene copy in the last common ancestor of Brassicales that was only retained by field mustard (Fig 3). The other copy was retained as a single copy gene in lyrate rockcress and thale cress, whereas several rounds of gene duplication in the field mustard gave rise to a repertoire of 4 CHS genes (Fig 3). The brown clade possesses species representation of the orders Malvales (cacao), Malpighiales (poplar, castor bean and cassava) and Rosales (apple and wild strawberry) (Fig 3) and is characterized by the fact that half of the species (wild strawberry, apple, and poplar) independently gave rise to multiple gene copies, whereas the other half (cassava, castor bean and cacao) maintained a single gene copy (Fig 3). Interestingly, the pink clade represents the gene lineage with the most gene copies (Fig 3). This lineage has only been retained in three species of the order Fabales (Fig 3). Our results suggest that this CHS gene underwent a duplication event in the last common ancestor of the order Fabales between 97 and 57 mya, and both paralogs were later retained in all three species (Fig 3), subsequently, all species greatly expanded one of the lineages (Fig 3). Thus, the barrel medic and *Lotus japonicus* expanded one of the paralogs to 14 and 11 copies, respectively, whereas they remained at low copy number in the other lineage (Fig 3). In contrast, soybean expanded the complementary gene lineage to 11 copies, whereas the other (which the other two species expanded) only possessed 2 copies (Fig 3).

From a functional perspective, past evidence has suggested that the genes that belong to the pink clade are the most responsive to the UV-B response regulatory machinery [29, 30]. According to Shimizu *et al*., CHS5 and CHS6 soybean genes, which correspond to the copy located on chromosome 9 and 8-T1 and -T6 in this study, respectively, are clearly up-regulated in plants treated with UV-B light, suggesting that this gene clade may be involved in UV protection [30]. Additionally, global transcriptional analyses of phenylpropanoid pathway genes [31–33] and the genetic manipulation of CHS genes [34] have suggested that genes belonging to the pink clade are also involved in functions related to the pathogen defense response. In combination with our evolutionary analyses, this cumulus of evidence suggests that CHS genes in the pink clade perform key stress-associated roles. The dramatic expansion of this clade is consistent with previous evidence that gene families exhibiting medium to high duplication counts were often involved in pathogen defense [35]. Thus, it appears that gene duplication allowed for the increase in gene product by expanding the repertoire of genes related to environmental challenges, allowing gene family diversification and long-term evolutionary plasticity [36–39]

## Conclusions

In this study, we provided a comprehensive evolutionary analysis of the chalcone synthase gene family in flowering plants that included representative species of the orders Brassicales, Malvaceae, Malpighiales, Rosales and Fabales, providing insight into the mechanisms that gave rise to gene copy number variation. Our results show that the last common ancestor of the rosid clade possessed six CHS gene lineages that were differentially retained during the evolutionary history of the group. We also showed that one of the lineages disproportionately expanded in species belonging to the order Fabales. Based on the available literature, we suggest that this gene lineage possesses stress-related biological functions (e.g., UV light response, pathogen defense). We propose that the expansion of this clade would be the result of a selective pressure to increase the amount of enzymes involved in phenylpropanoid pathway-derived secondary metabolite production, consistent with the hypothesis proposed by Fischer *et al*. in which lineage-specific expansions fuel plant adaptation [39].

## Supporting Information

S1 File Nucleotide alignment. Nucleotide alignment used to reconstruct the best tree depicted in Fig 3.
(TXT)

S2 File Evolutionary models. Evolutionary models used for the phylogenetic analyses using treefinder.
(TXT)

S3 File Best tree. Best tree topology inferred using a maximum likelihood approach in treefinder.
(TXT)

S1 Table Accession numbers used in this study. Scientific names, genomic location and accession numbers of the chalcone synthase genes used in this study.
(XLSX)

## References

[1] Ohno S. Evolution by gene duplication. Berlin, New York,: Springer-Verlag; 1970.

[2] Force A, Lynch M, Pickett FB, Amores A, Yan YL, Postlethwait J. Preservation of duplicate genes by complementary, degenerative mutations. Genetics. 1999;151(4):1531–45. ; PMCID: PMCPMC1460548. 10101175 10.1093/genetics/151.4.1531 PMC1460548

[3] Vanneste K, Baele G, Maere S, Van de Peer Y. Analysis of 41 plant genomes supports a wave of successful genome duplications in association with the Cretaceous–Paleogene boundary. Genome Res. 2014;24(8):1334–47. 10.1101/gr.168997.113 24835588 PMC4120086

[4] Wood TE, Takebayashi N, Barker MS, Mayrose I, Greenspoon PB, Rieseberg LH. The frequency of polyploid speciation in vascular plants. Proc. Nat. Acad. Sci. USA. 2009;106(33):13875–9. 10.1073/pnas.0811575106 19667210 PMC2728988

[5] Judd WS, Campbell CS, Kellogg EA, Stevens PF, Donoghue MJ. Plant systematics: a phylogenetic approach. Sunderland, Massachusetts, USA.

[6] Soltis DE, Soltis PE, Endress PK, Chase MW. Phylogeny and Evolution of Angiosperms. First ed Sunderland, MA: Sinauer Associates, Inc.; 2005.

[7] Magallon S, Crane PR, Herendeen PS. Phylogenetic Pattern, Diversity, and Diversification of Eudicots. Ann. Miss. Bot. Gar. 1999 p. 292–372.

[8] Wang H, Moore MJ, Soltis PS, Bell CD, Brockington SF, Alexandre R, et al Rosid radiation and the rapid rise of angiosperm-dominated forests. Proc. Nat. Acad. Sci. USA. 2009 10.1073/pnas.0813376106 PMC2644257 19223592

[9] Harborne JB. The Flavonoids: Advances in Research Since 1986. Journal of Chemical Education. 1995;72(3):A73 10.1021/ed072pA73.11

[10] Ferreyra M, Rius S, Casati P. Flavonoids: biosynthesis, biological functions, and biotechnological applications. Front. Plant Sci. 2012;3 10.3389/fpls.2012.00222 WOS:000208837900219. PMC3460232 23060891

[11] Flores-Sanchez IJ, Verpoorte R. Plant polyketide synthases: a fascinating group of enzymes. Plant Physiol Biochem. 2009;47(3):167–74. 10.1016/j.plaphy.2008.11.005 . 19071029

[12] Harborne JB. The Flavonoids: advances in research since 1980. London; New York: Chapman and Hall; 1988.

[13] Lanz T, Tropf S, Marner FJ, Schröder J, Schröder G. The role of cysteines in polyketide synthases. Site-directed mutagenesis of resveratrol and chalcone synthases, two key enzymes in different plant-specific pathways. J Biol Chem. 1991;266(15):9971–6. . 2033084

[14] Helariutta Y, Elomaa P, Kotilainen M, Griesbach RJ, Schroder J, Teeri TH. Chalcone synthase-like genes active during corolla development are differentially expressed and encode enzymes with different catalytic properties in Gerbera hybrida (Asteraceae). Plant Mol Biol. 1995;28(1):47–60. Epub 1995/04/01. . 7787187 10.1007/BF00042037

[15] Ferrer JL, Jez JM, Bowman ME, Dixon RA, Noel JP. Structure of chalcone synthase and the molecular basis of plant polyketide biosynthesis. Nat Struct Biol. 1999;6(8):775–84. 10.1038/11553 . 10426957

[16] Feinbaum R, Ausubel F. Transcriptional regulation of the Arabidopsis thaliana chalcone synthase gene. Mol. Cell Biol. 1988;8(5):1985–92. WOS:A1988N192000015. 3386631 10.1128/mcb.8.5.1985-1992.1988 PMC363377

[17] Forkman G. Genetics of flavonoids In: Harborne JB, editor. The Flavonoids: Advances in Research since 1986. London: Chapman and Hall; 1993 p. 583–64.

[18] Tatusova T, Madden T. BLAST 2 SEQUENCES, a new tool for comparing protein and nucleotide sequences. Fems Microbiol. Lett. 1999;174(2):247–50. 10.1111/j.1574-6968.1999.tb13575.x WOS:000080230000006. 10339815

[19] Jobb G, von Haeseler A, Strimmer K. TREEFINDER: a powerful graphical analysis environment for molecular phylogenetics. BMC Evol Biol. 2004;4:18 10.1186/1471-2148-4-18 ; PMCID: PMCPMC459214. 15222900 PMC459214

[20] Gil M, Zanetti MS, Zoller S, Anisimova M. CodonPhyML: fast maximum likelihood phylogeny estimation under codon substitution models. Mol Biol Evol. 2013;30(6):1270–80. 10.1093/molbev/mst034 ; PMCID: PMCPMC3649670. 23436912 PMC3649670

[21] Katoh K, Asimenos G, Toh H. Multiple Alignment of DNA Sequences with MAFFT. Methods Mol. Biol. 537:39–64. 10.1007/978-1-59745-251-9_3 19378139

[22] Abascal F, Zardoya R, Telford MJ. TranslatorX: multiple alignment of nucleotide sequences guided by amino acid translations. Nucleic Acids Res. 2010;38(Web Server issue):W7–13. 10.1093/nar/gkq291 ; PMCID: PMCPMC2896173. 20435676 PMC2896173

[23] Goldman N, Yang Z. A codon-based model of nucleotide substitution for protein-coding DNA sequences. Mol. Biol. Evol. 1994;11(5):725–36. 7968486 10.1093/oxfordjournals.molbev.a040153

[24] Anisimova M, Gil M, Dufayard JF, Dessimoz C, Gascuel O. Survey of branch support methods demonstrates accuracy, power, and robustness of fast likelihood-based approximation schemes. Syst Biol. 2011;60(5):685–99. 10.1093/sysbio/syr041 ; PMCID: PMCPMC3158332. 21540409 PMC3158332

[25] FukadaTanaka S, Hoshino A, Hisatomi Y, Habu Y, Hasebe M, Iida S. Identification of new chalcone synthase genes for flower pigmentation in the Japanese and common morning glories. Plant Cell Physiol. 1997;38(6):754–8. WOS:A1997XF27600017. 9249990 10.1093/oxfordjournals.pcp.a029232

[26] McKhann HI, Hirsch AM. Does Rhizobium avoid the host response? Curr Top Microbiol Immunol. 1994;192:139–62. Epub 1994/01/01. . 7859504 10.1007/978-3-642-78624-2_7

[27] Akada S, Dube SK. Organization of soybean chalcone synthase gene clusters and characterization of a new member of the family. Plant Mol Biol. 1995;29(2):189–99. . 7579172 10.1007/BF00043645

[28] Lynch M. The frailty of adaptive hypotheses for the origins of organismal complexity. Proc. Nat. Acad. Sci. USA. 2007;104(suppl 1):8597–604. 10.1073/pnas.0702207104 17494740 PMC1876435

[29] Akada S, Kung SD, Dube SK. Nucleotide sequence of a soybean chalcone synthase gene with a possible role in ultraviolet-B sensitivity, Gmchs6. Plant Physiol. 1993;102(2):699–701. 8108524 10.1104/pp.102.2.699 PMC158838

[30] Shimizu T, Akada S, Senda M, Ishikawa R, Harada T, Niizeki M, et al Enhanced expression and differential inducibility of soybean chalcone synthase genes by supplemental UV-B in dark-grown seedlings. Plant Mol Biol. 1999;39(4):785–95. . 10350092 10.1023/a:1006124219945

[31] Dakora FD, Phillips DA. Diverse functions of isoflavonoids in legumes transcend anti-microbial definitions of phytoalexins. Physiol. Mol. Plant P. 1996;49(1):1–20. 10.1006/pmpp.1996.0035

[32] Dixon RA, Steele CL. Flavonoids and isoflavonoids—a gold mine for metabolic engineering. Trends Plant Sci. 1999;4(10):394–400. . 10498963 10.1016/s1360-1385(99)01471-5

[33] Zabala G, Zou J, Tuteja J, Gonzalez DO, Clough SJ, Vodkin LO. Transcriptome changes in the phenylpropanoid pathway of Glycine max in response to Pseudomonas syringae infection. BMC Plant Biol. 2006;6:26 10.1186/1471-2229-6-26 ; PMCID: PMCPMC1636052. 17083738 PMC1636052

[34] Lozovaya VV, Lygin AV, Zernova OV, Ulanov AV, Li S, Hartman GL, et al Modification of phenolic metabolism in soybean hairy roots through down regulation of chalcone synthase or isoflavone synthase. Planta. 2007;225(3):665–79. 10.1007/s00425-006-0368-z . 16924535

[35] Cannon S, Mitra A, Baumgarten A, Young N, May G. The roles of segmental and tandem gene duplication in the evolution of large gene families in Arabidopsis thaliana. BMC Plant Biology. 2004;4:10 10.1186/1471-2229-4-10 15171794 PMC446195

[36] Baumgarten A, Cannon S, Spangler R, May G. Genome-level evolution of resistance genes in Arabidopsis thaliana. Genetics. 2003;165(1):309–19. ; PMCID: PMCPMC1462749. 14504238 10.1093/genetics/165.1.309 PMC1462749

[37] Leister D. Tandem and segmental gene duplication and recombination in the evolution of plant disease resistance gene. Trends Genet. 2004;20(3):116–22. . 15049302 10.1016/j.tig.2004.01.007

[38] Meyers BC, Kaushik S, Nandety RS. Evolving disease resistance genes. Curr Opin Plant Biol. 2005;8(2):129–34. 10.1016/j.pbi.2005.01.002 . 15752991

[39] Fischer I, Dainat J, Ranwez V, Glemin S, Dufayard J, Chantret N. Impact of recurrent gene duplication on adaptation of plant genomes. BMC Plant Biol. 2014;14 10.1186/1471-2229-14-151 WOS:000338158700002. PMC4049390 24884640

